# Multisensory perception as an associative learning process

**DOI:** 10.3389/fpsyg.2014.01095

**Published:** 2014-09-26

**Authors:** Kevin Connolly

**Affiliations:** Philosophy and Institute for Research in Cognitive Science, University of PennsylvaniaPhiladelphia, PA, USA

**Keywords:** perceptual learning, crossmodal integration, feature binding, multimodal interaction, crossmodal interaction, binding, multisensory integration, associative learning

## Abstract

Suppose that you are at a live jazz show. The drummer begins a solo. You see the cymbal jolt and you hear the clang. But in addition seeing the cymbal jolt and hearing the clang, you are also aware that the jolt and the clang are part of the same event. Casey O’Callaghan (forthcoming) calls this awareness “intermodal feature binding awareness.” Psychologists have long assumed that multimodal perceptions such as this one are the result of a automatic feature binding mechanism (see Pourtois et al., 2000; Vatakis and Spence, 2007; Navarra et al., 2012). I present new evidence against this. I argue that there is no automatic feature binding mechanism that couples features like the jolt and the clang together. Instead, when you experience the jolt and the clang as part of the same event, this is the result of an associative learning process. The cymbal’s jolt and the clang are best understood as a single learned perceptual unit, rather than as automatically bound. I outline the specific learning process in perception called “unitization,” whereby we come to “chunk” the world into multimodal units. Unitization has never before been applied to multimodal cases. Yet I argue that this learning process can do the same work that intermodal binding would do, and that this issue has important philosophical implications. Specifically, whether we take multimodal cases to involve a binding mechanism or an associative process will have impact on philosophical issues from Molyneux’s question to the question of how active or passive we consider perception to be.

## INTRODUCTION

Suppose that you are at a live jazz show. The drummer begins a solo. You see the cymbal jolt and you hear the clang. But in addition seeing the cymbal jolt and hearing the clang, you are also aware that the jolt and the clang are part of the same event. Casey O’Callaghan (forthcoming) calls this awareness “intermodal feature binding awareness.” It is *intermodal*, meaning that it involves more than one sense modality. It is *feature binding* in that the features are perceived as jointly bound to the same object or event. And it is *awareness*, because you are conscious of the features being bound to the object or event in this way.

While I agree that we can have awareness that the jolt and the clang are part of the same event, I will argue that there is no automatic feature binding mechanism that binds features like the jolt and the clang together. Instead, when you experience the jolt and the clang as part of the same event, this is the result of an associative learning process. The cymbal’s jolt and the clang are best understood as a single learned perceptual unit, rather than as automatically bound. More generally, my claim is that multimodal cases involve learned associations, and I will outline a specific learning process in perception whereby we come to “chunk” the world into multimodal units. A central contribution of the paper is this: unitization is an entirely undiscussed way that an associationist might implement an associative account of multimodal perception. It is one thing to say that features *x* and *y* are associated. It is another thing to give a detailed account (drawing on an established perceptual learning process) of how exactly that association happens. In what follows, I attempt to do exactly that.

It can be difficult to tease apart the difference between an account of multimodal perception based on intermodal feature binding and an account based on associative learning. For now, the key question to ask is how features, such as a jolt and a clang, come to be coupled. Specifically, did the coupling happen in past experience, or did it happen just prior to your current experience? In other words, if you experience a jolt and a clang as part of the same event, is this due to those features getting coupled in your past experience, or did the coupling of the jolt and the clang occur just prior to your experience of them?

If feature binding awareness does not involve feature binding—which is what I will argue—then this flies in the face of the way that scientists working on multimodal perception have been thinking about these cases. Consider four such representative passages highlighted by O’Callaghan(forthcoming, ms pp. 8–9):

When presented with two stimuli, one auditory and the other visual, an observer can perceive them either as referring to the same unitary audiovisual event or as referring to two separate unimodal events .... *There appear to be specific mechanisms in the human perceptual system involved in the binding of spatially and temporally aligned sensory stimuli.* (Vatakis and Spence, 2007, 744, 754, italics were added for emphasis).

As an example of such privileged binding, we will examine the relation between visible impacts and percussive sounds, which allows for *a particularly powerful form of binding that produces audio-visual objects*. (Kubovy and Schutz, 2010, 42, italics were added for emphasis).

In a natural habitat information is acquired continuously and simultaneously through the different sensory systems. As some of these inputs have the same distal source (such as the sight of a fire, but also the smell of smoke and the sensation of heat) it is reasonable to suppose that the organism should be able to bundle or bind information across sensory modalities and not only just within sensory modalities. For one such area where intermodal binding (IB) seems important, that of concurrently seeing and hearing affect, *behavioural studies have shown that indeed intermodal binding takes place during perception.* (Pourtois et al., 2000, 1329, italics were added for emphasis).

[T]here is undeniable evidence that the visual and auditory aspects of speech, when available, contribute to an integrated perception of spoken language .... *The binding of AV speech streams seems to be, in fact, so strong* that we are less sensitive to AV asynchrony when perceiving speech than when perceiving other stimuli (Navarra et al., 2012, 447, italics were added for emphasis)^1^.

The traditional view is that multimodal perception at the conscious-level is the result of intermodal feature binding at the unconscious-level in all the ways mentioned above, whether it is with spatially and temporally aligned stimuli, audio-visual objects, with facial expression and tone of voice, or audio-visual speech streams. I will argue that this view is mistaken.

Whether multimodal perception involves an automatic binding process or an associative process has been discussed before (in the case of speech perception, for instance, see Altieri and Townsend, 2011; Altieri et al., 2011). Starting with the former view, the theory that multimodal perception involves an automatic binding process is consistent with several other theories in the psychological literature on perception, including Gibson’s (1950, 1972, 1979) theory of direct perception and Fowler’s discussion of speech as an amodal phenomenon (Fowler, 2004). On Gibson’s view, for instance, we directly perceive objects with their features already integrated. We do not have to associate the jolt and the clang, for instance, because we directly perceive the cymbal, and the jolt and clang features are already integrated into the cymbal. Similarly, Fowler (2004) discusses the view that listeners directly perceive speech gestures. A gestural percept is amodal, as she describes it, with information from different sense modalities already integrated into it. On this view, you perceive a speech gesture with the auditory and visual features already integrated into it.

**FIGURE 1:**
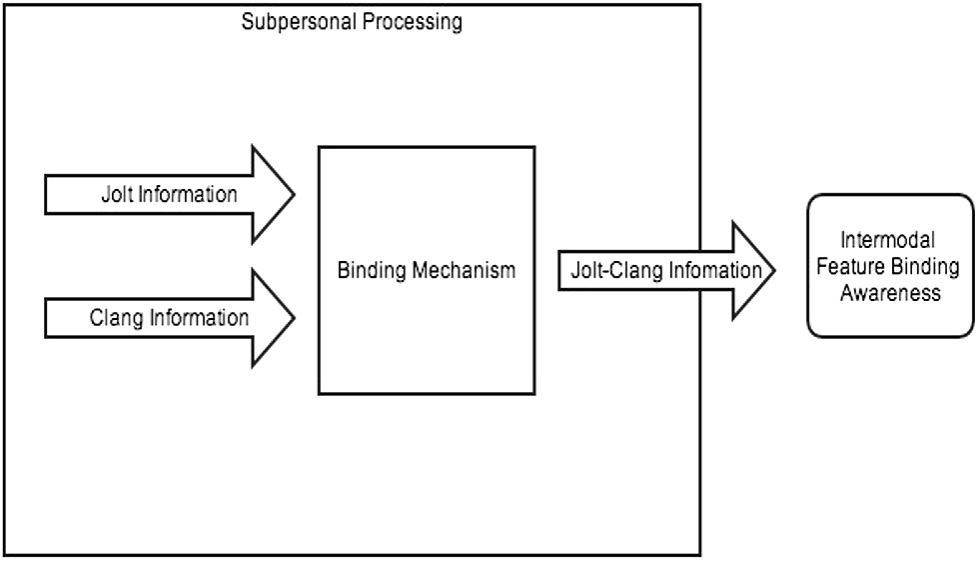
**Multimodal perception as an automatic binding process**.

What Gibson, Fowler, and the binding view have in common is that features are automatically bound outside of and prior to conscious perception. Since processing happens early, a good model is a coactive model, which Townsend and Nozawa (1995) define as, “A parallel architecture which assumes that input from the separate parallel channels is consolidated into a resultant common processor” (p. 323). The binding mechanism, in this case, would serve as the common processor that consolidates information channels from different sense modalities. On a coactive model, the jolt and the clang information would be consolidated into the binding mechanism, and the output of that mechanism results in those bound features being available to consciousness. This enables awareness that the features are part of the same event (see **Figure 1**).

The view that multimodal perception results from an associative learning process, on the other hand, is consistent with several other theories in the psychological literature on perception. Smith and Yu (2007, 2008), for example, have studied how both infants and adults match words to scenes. As Quine (1960) pointed out, given a somewhat complex scene, for any given word, there are an infinite number of possible referents. Yet, Smith and Yu (2007, 2008) and Yu and Smith (2006, 2007, 2011, 2012) show how the binding of a word and a referent occurs through an associative learning process whereby infants and adults learn, across varying contexts, the statistical likelihood that a word refers to a particular kind of object). Along the same lines, Wallace (2004) describes how multisensory neurons require a protracted maturation process. Specifically, multisensory neurons get strengthened through experience.

**FIGURE 2:**
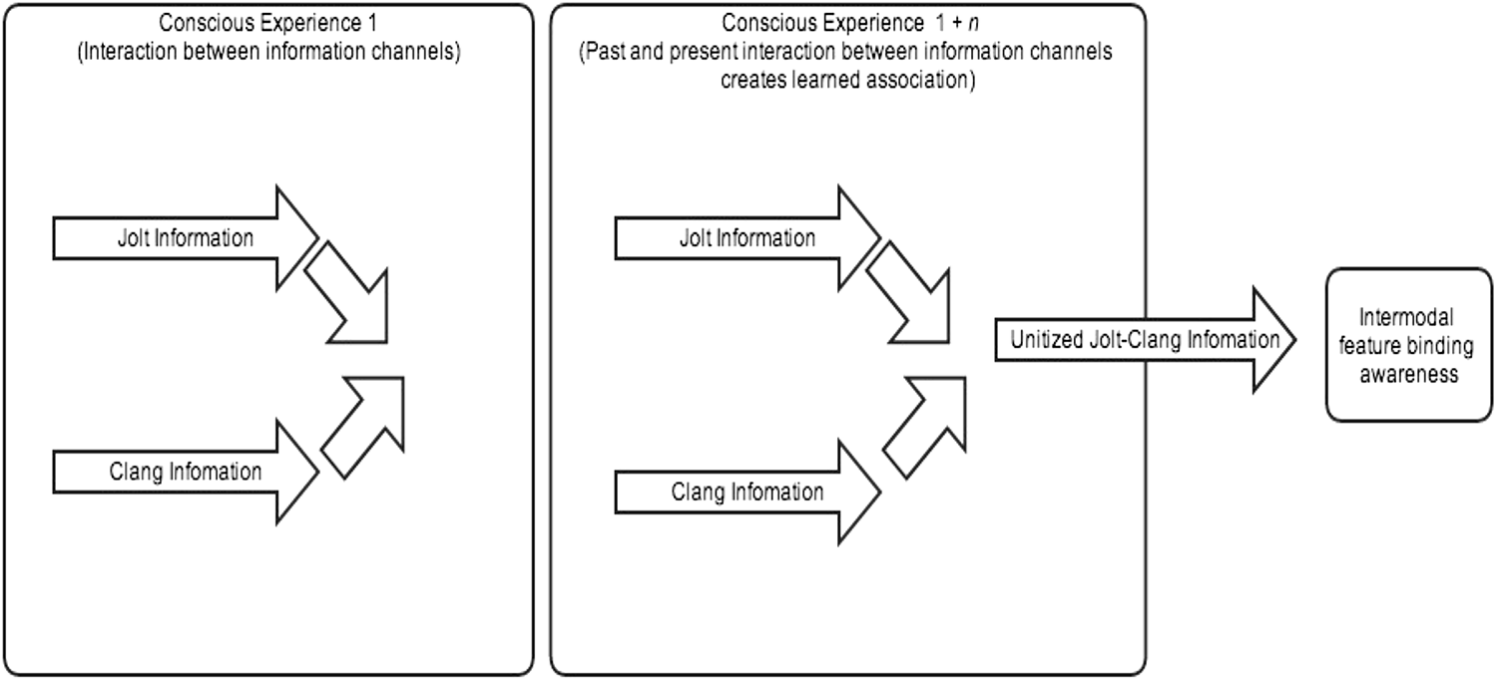
**Multimodal perception as an associative learning process**.

What holds the theories of Smith and Yu, Wallace, and myself in common is that on our views features are not coupled by an automatic mechanism, but rather get coupled through an associative learning process. While associative accounts have been offered for speech perception, as Smith and Yu do, they have rarely been applied to multimodal cases outside of speech perception, and this paper shows how they can be applied in that way. A good model for associative learning is an interactive parallel processing model (see Townsend and Wenger, 2004). According to this model, the information channels from different sense modalities are processed in parallel, but can interact. The jolt and the clang information, for instance, involve parallel processing, and the interaction between the two information streams enables them to become associated. The result of this association is that the jolt and the clang are later experienced, not as distinct, but as part of one and the same event (see **Figure 2**)^2^.

My plan for the rest of the paper is as follows. In section “Why is the Debate Significant?” I will say why it is significant whether we take multimodal cases to involve a binding mechanism or an associative process at the unconscious-level. In particular, it will have impact on philosophical issues from Molyneux’s question to the question of how active or passive we should consider perception to be. In section “Intermodal Feature Binding Awareness,” I will briefly explain O’Callaghan’s notion of intermodal feature binding awareness—an account that details what is happening at the conscious-level for cases that many psychologists have taken to involve intermodal feature binding at the unconscious-level. In section “Unitization,” I will offer a previously undiscussed alternative to intermodal feature binding—what’s called “unitization” in the literature on perceptual learning. In section “Applying Unitization to Multimodal Cases,” I will apply unitization to multimodal cases, and show how the phenomenon is consistent with O’Callaghan’s main argument for intermodal feature binding awareness. In section “Objections and Responses,” I will respond to some objections.

## WHY IS THE DEBATE SIGNIFICANT?

Why does it matter whether intermodal feature binding awareness is the result of intermodal binding or of learned associations? One reason has to do with the implications the issue has for a long-standing philosophical problem. Molyneux’s question asks whether a man born blind, who can distinguish a cube and sphere by touch, could distinguish those shapes upon having his sight restored. If intermodal feature binding awareness is the result of learned associations, then we have a straightforward “no” answer to Molyneux’s question. You see the cube for the first time. No learned associations have taken place between sight and touch. So, no, you don’t recognize which is the cube and which is the sphere (see **Table 1**).

**Table 1:** Multimodal perception: associative learning vs. intermodal feature binding.

	Associative learning	Intermodal feature binding
*When does the coupling happen?*	In past experience	Jut prior to present experience
*Can the theory accommodate intermodal feature binding awareness?*	Yes (see “Applying Unitization to Multimodal Cases”)	Yes
*How does the theory answer Molyneux’s question?*	Definitive “No”	Likely “Yes”
*What kind of processing model fits the Theory?*	Interactive parallel processing model	Coactive model

How we answer Molyneux’s question will, in turn, have ramifications for debates between nativists or rationalists such as Leibniz (1765/1982), on the one hand, and empiricists such as Locke (1690/1975) on the other hand. On Molyneux’s question, nativists hold that the association between the felt and seen cube is innate, while empiricists hold that it is learned. If the association between the felt and seen cube is learned, therefore yielding a “no” answer to Molyneux’s question, then this gives us an empiricist answer to Molyneux’s question, rather than a nativist one.

Recent experimental evidence lends support to the claim that the answer to Molyneux’s question is a “no.” A study conducted by Held et al. (2011) tested whether subjects who had just undergone cataract removal surgery for sight restoration, would be able to identify previously felt legos by sight. In the study, subjects first were given one lego to touch. Next, they were visually presented with two distinctly shaped legos, and were asked which of the two legos they had previously been touching. Subjects performed at near-chance levels in answering this question. Held and colleagues interpret this result to mean that the answer to Molyneux’s question is likely to be “no,” since subjects born blind, who could distinguish between shapes by touch, could not distinguish those shapes upon having their sight restored (for a debate about the experimental design in Held et al., 2011; see Schwenkler, 2012, 2013; Connolly, 2013).

A second reason it matters whether intermodal feature binding awareness is the result of intermodal binding or of learned associations is that, as O’Callaghan has pointed out, one of the most important discoveries in the cognitive science of perception in the past two decades is that the senses involve extensive interaction and coordination. We want to understand how this works, and many cases of multisensory awareness are cases of binding awareness. But are cases of binding awareness the result of intermodal binding, or are they the result of learned associations? Depending on which one of these is our answer, we will have a different account of one of the most important discoveries in the cognitive science of perception in the past two decades.

A third reason the debate is important is that a view that makes multimodal perception a flexible, learned process (see, for instance, Connolly, 2014) fits more naturally with the emerging view of perception as a more active process than it has typically been taken to be. That is to say, it fits with a view of perception where the perceiver works to construct the world through learning and exploration rather than just passively receiving inputs that get transformed into a representation of the world. On an active view the perceiver does not simply look at the world as a passive observer, but has to look around, explore, and tweak the processes that are involved in perception to make them more useful to them for knowing what is out in the world, and for interacting with the world in an effective way.

## INTERMODAL FEATURE BINDING AWARENESS

When you are listening to the drum solo, see the cymbal jolt, hear the clang, and are also aware that the jolt and the clang are part of the same event, this is a case of intermodal feature binding awareness. Why is intermodal feature binding awareness of theoretical significance? One reason is that it propels an argument, made by O’Callaghan (2014), that not all perceptual experience is modality specific, that is to say, that there are cases of multimodal perception which cannot be broken down into just seeing, hearing, touching, tasting, and smelling, happening at the same time. As he puts it, perceptual awareness is not just “minimally multimodal.” It is not just exhausted by perceptual awareness in each of the sense modalities happening at the same time.

Why does O’Callaghan deny minimal multimodality? One reason is due to intermodal feature binding awareness. Intermodal feature binding awareness occurs when you consciously perceive multiple features from more than one sense modality jointly to belong to the same object or event. O’Callaghan’s main argument for intermodal feature binding awareness runs as follows. Consider the difference between the following cases one and two. In case one, when the drummer begins a solo, you see the cymbal jolt and hear the clang, and you are aware that the jolt and the clang are part of the same event. In case two, you see the jolt and hear the clang, but you are not aware that the jolt and the clang are part of the same event. Perhaps you have never seen a cymbal before and are unaware of the sound that it makes. According to O’Callaghan, there may be a phenomenal difference between case one and case two. This difference is explicable in terms of intermodal feature binding awareness: case one involves such an awareness, while case two does not. O’Callaghan generalizes the point: “a perceptual experience as of something’s being F and G may differ in phenomenal character from an otherwise equivalent perceptual experience as of something F and something G, where F and G are features perceptually experienced through different modalities” (O’Callaghan, 2014, ms p. 8). This is just to say that in the cymbal example and others like it, case one differs from case two in terms of its phenomenology. O’Callaghan explains this difference in that the former, but not the latter case involves intermodal feature binding awareness.

Everything said so far is about feature binding *awareness*. This is something that happens at the conscious-level. But psychologists often talk about feature binding, and there they are referring to a unconscious process. As a representative view, Vatakis and Spence claim:

When presented with two stimuli, one auditory and the other visual, an observer can perceive them either as referring to the same unitary audiovisual event or as referring to two separate unimodal events .... *There appear to be specific mechanisms in the human perceptual system involved in the binding of spatially and temporally aligned sensory stimuli.* (Vatakis and Spence, 2007, 744, 754; quoted by O’Callaghan, 2014, ms p. 8)

But what is the connection between feature binding awareness and the feature binding process? The assumption in the empirical literature is that cases like the cymbal case depend upon feature binding at the unconscious-level—an assumption that I will argue is mistaken. Roughly and briefly, on my view, cases like the cymbal case are best explained through a process called “unitization,” whereby features (such as the jolt and the clang) that were once detected separately, are later detected as a single unit. For example, while someone who has never seen a cymbal before might plausibly experience the clash and the jolt not as the part of the same event, others unitize those features into the same event, due to learning.

O’Callaghan’s own argument is about feature binding *awareness*, which he describes as likely related to—but not the same as—feature binding itself. O’Callaghan explains the connection: “Feature binding awareness presumably depends upon feature binding processes. I say “presumably” because a feature binding process ... may require that features are detected or analyzed separately by subpersonal perceptual mechanisms” (forthcoming, ms p. 3). At the same time, O’Callaghan distances himself from feature binding processes. He allows that “it is possible that what I have characterized as feature binding awareness could occur without such a feature binding process” (forthcoming, ms p. 3). So, on O’Callaghan’s view, the existence of feature binding awareness does not imply a feature binding process.

O’Callaghan’s account of feature binding awareness is consistent with my view, since I deny a feature binding process, and his view does not imply such a process. But one place where O’Callaghan and I differ is with the name “*feature binding* awareness.” If there is an associative process involved rather than a feature binding mechanism and the result of the associative process manifests itself at the conscious-level (and I will argue that this is the case), it is hard to see why we should call the conscious upshot “feature binding awareness” rather than “associative awareness.” If there is an associative process, then since we will have ruled out a feature binding mechanism in favor of a different process, it would be inaccurate to call the conscious upshot “feature binding awareness.^3^”

At the same time, O’Callaghan and I are united in our departure from Spence and Bayne (2014), who say the following:

But are features belonging to different modalities bound together in the form of MPOs [multimodal perceptual objects]? ... [W]e think it is debatable whether the “unity of the event” really is internal to one’s experience in these cases, or whether it involves a certain amount of post-perceptual processing (or inference). In other words, it seems to us to be an open question whether, in these situations, one’s experience is of a MPO or whether instead it is structured in terms of multiple instances of unimodal perceptual objects. (Spence and Bayne, 2014, ms 27, 29; quoted by O’Callaghan, forthcoming, p. 5)

On Spence and Bayne’s account, it is debatable whether intermodal feature binding awareness occurs at all. So, in the cymbal case, where O’Callaghan and I think that you can see the cymbal jolt and hear the clang, and be aware that the jolt and the clang are part of the same event, Spence and Bayne think that is debatable. One alternative, they might say, is that you see the jolt of the cymbal, hear the clang, and infer that they are both associated with the same object. And on their view, it is an open question whether such an alternative is correct.

O’Callaghan’s account is restricted to the conscious-level. But we can ask what the unconscious processes are which produce it. Psychologists have assumed that intermodal feature binding produces multimodal perception, but I will now explore a previously undiscussed alternative to intermodal feature binding—what is called “unitization” in the literature on perceptual learning.

## UNITIZATION

Robert Goldstone, one of the leading psychologists working on perceptual learning today, lists unitization as one of four mechanisms of *perceptual learning*. What is perceptual learning? Eleanor Gibson defines it as “any relatively permanent and consistent change in the perception of a stimulus array, following practice or experience with this array” (Gibson, 1963, p. 29). Perceptual learning involves perceptual changes. Perceptual changes occur so that we can better perform the cognitive tasks that we need to do. The idea is that to ideally perform cognitive tasks, it is better for perceptual systems to be flexible, rather than hardwired. As Goldstone puts it, one might be tempted to hold that the perceptual system is hardwired, the intuition being that “stable foundations make strong foundations” (Goldstone, 2010, p. v). But actually a better model of perception is a suspension bridge: “Just as a suspension bridge provides better support for cars by conforming to the weight loads, perception supports problem solving and reasoning by conforming to these tasks” (Goldstone, 2010, p. v). Perceptual systems are flexible rather than hardwired so that they can better support cognitive tasks. Specifically, the kind of flexibility on which I will focus is how perceptual systems are able to construct perceptual units of the various different sizes, which improve our ability to respond to our environment.

When people hear about perceptual learning, they often think of cases of improved discrimination abilities. William James, for instance, writes of a man who has learned to distinguish by taste between the upper and lower half of a particular type of wine (James, 1890, p. 509). What the man’s perceptual system had previously treated as a single thing is later treated as two distinct things. Psychologists who work on perceptual learning call this *differentiation*. But the converse happens as well. Sometimes, what has been treated previously by the perceptual system as two things, is later treated by it as one thing. Psychologists call this *unitization*. Perceptual units are created not just by breaking down larger units (like the bottle of wine) into smaller one’s (like the top half and the bottom half), but also by merging smaller units into larger ones.

As Goldstone puts it, “Unitization involves the construction of single functional units that can be triggered when a complex configuration arises. Via unitization, a task that originally required detection of several parts can be accomplished by detecting a single unit .... [U]nitization integrates parts into single wholes” (Goldstone, 1998, p. 602)^4^. For example, consider someone who is developing an expertise in wine tasting and is learning to detect Beaujolais. Detecting it at first might involve detecting several features, such as the sweetness, tartness, and texture. But detecting the Beaujolais is later accomplished by just detecting it as a single unit. Since the Beaujolais gets unitized by your perceptual system, this allows you to quickly and accurately recognize it, when you taste it.

According to Goldstone and Byrge, unitization in perception is akin to “chunking” in memory (Goldstone and Byrge, 2014, ms p. 15). Normally, we are only able to commit 7±2 items into short-term memory. Yet, we are easily able to do much better with the following string of 27 letters, by chunking them:

M O N T U E W E D F B I C I A K G B C B S N B C A B C

We can chunk the first nine letters as abbreviations for days of the week, the next nine as abbreviations for intelligence agencies, and the final nine as abbreviations for American television networks. Chunking is the building of new units that help to enable memory. Similarly, in perception, unitization allows us to encode complex information, which without unitization we might be unable to encode. Suppose, for instance, that you are drinking an extremely complex Beaujolais that you have never tasted before. Your perceptual system might unitize that type of wine, allowing you to recognize it, despite the fact that it is extremely complex.

A whole host of objects have been shown to be first processed as distinct parts, and later processed as a unit. Goldstone and Byrge offer the following diverse list: “birds, words, grids of lines, random wire structures, fingerprints, artificial blobs, and 3-D creatures made from simple geometric components” (Goldstone and Byrge, 2014, ms p. 17). Unitization occurs not just for things like cats and cars, but also for objects constructed in the lab. For instance, Gauthier and Tarr (1997) constructed a set of objects called “Greebles,” which shared a set of spatial features in common. When the subjects were exposed to the Greebles for long enough, they would begin to process them as units (Gauthier and Tarr, 1997). This showed up in the fact that people trained with the Greebles performed better than novices on speed and accuracy tests.

Many of the cases mentioned so far involve parts being treated as wholes after unitization, as when parts of a Greeble get treated as a whole unit. However, there are also cases in which attributes or properties become treated as units. For instance, a study by Shiffrin and Lightfoot (1997) showed that subjects are able to unitize the angular properties (i.e., *horizontality*, *verticality*, or *diagonality*) of a set of line segments. The study involved sets of three line segments, each of the segments angled either horizontally, vertically, or diagonally. Subjects were given a target set. Say, for instance, that the target set is a set of two horizontal and one vertical line segments. Given that target, the subjects were asked to pick matching targets out (that is, all and only sets involving two horizontal and one vertical line segment), and ignore distractors (such as a set of two vertical and one horizontal line segments, or three horizontal line segments, among others). Subjects became very quick at this task through training, indicating that they had unitized the angular attributes of each of the three line segments.

As objects become unitized, the whole becomes easier to process perceptually than the part. At first, when one is learning what Greebles are, it is essential to identify them by their features. After they become unitized, however, it is easier to process them as whole units. Similarly, faces are unitized—they are easier to process as wholes than as parts. One interesting feature of face unitization is that inverting a face disrupts the unitization process. This means that faces are harder to recognize when presented upside-down than when presented right-side up (Diamond and Carey, 1986). Furthermore, if you distort features of a face, the distortions are quite apparent when the face is right-side up, but much less apparent when the face is upside-down. This effect, called the *Thatcher effect*, seems to show something important about the phenomenology of a unitized object. Specifically, what it is like to experience the upside-down distorted face is not simply what it is like to experience the right-side up distorted face plus inversion. Rather, there is something that it is like to experience, say, a distorted nose and lips in a unitized face, and that is different from what it is like to experience a distorted nose and lips in a non-unitized face.

## APPLYING UNITIZATION TO MULTIMODAL CASES

My claim is that we unitize things, sometimes unimodally, as in the case of faces, birds, grids of lines, random wire structures, artificial blobs, and fingerprints. But sometimes unitization occurs multimodally as well. As Goldstone writes, “Neural mechanisms for developing configural units with experience are located in the superior colliculus and inferior temporal regions. Cells in the superior colliculus of several species receive inputs from many sensory modalities (e.g. visual, auditory, and somatosensory), and differences in their activities reflect learned associations across these modalities” (Goldstone, 1998). So, unitization occurs in part in the superior colliculus, a place that in cats and macaque monkeys receives multisensory inputs (see Stein and Wallace, 1996, p. 290).

Reconsider the difference between case one and case two of the cymbal example. In case one, you see the jolt of the cymbal, hear the clang, and are aware that the jolt and the clang are part of the same event. In case two, you see the jolt and hear the clang, but are not aware that they are part of the same event. My claim is that in case one, the jolt and the clang are unitized in the same event, while in case two they are not. Interestingly enough, one reason why case two might occur in the first place is if you have never seen a cymbal before, and so you have not built the association between what a cymbal looks like when it has been struck and what it sounds like.

This gives us a substantive reply to O’Callaghan’s main argument for intermodal feature binding awareness. O’Callaghan argues for intermodal feature binding awareness by distinguishing between intermodal cases (1) and (2):

(1) Perceiving a thing’s being both *F* and *G* (where *F* and *G* are features that are perceived through different sense modalities).
(2) Perceiving a thing’s being *F* and a thing’s being *G.*

His idea is that (1) involves intermodal feature binding awareness, while (2) does not. But what I am saying is that the difference between (1) and (2) does not entail that intermodal feature binding has occurred (as psychologists have argued). We can distinguish between (1) and (2) phenomenally without appealing to intermodal feature binding. If (1) involves unitization, while (2) does not, then the phenomenal difference between them is that in (1), *F* and *G* are unitized in the thing, while in (2), they are not unitized in the thing.

Put more formally, in the case where you see the cymbal jolt and you hear the clang, let E1[f(x)] and E2[g(y)] denote that seeing the jolt *x* is a function *f* of vision and that the jolt is experienced as part of event 1, while hearing the clang *y* is a function *g* of audition and the clang is experienced as part of event 2. This is case one. Let E1[f(x), g(y)] denote that seeing the jolt *x* is a function *f* of vision and the jolt is experienced as part of event one, while hearing the clang *y* is a function *g* of audition and the clang is also experienced as part of event one. This is case two, which is distinct from case one in that case two involves a single event while case one involves two events^5^.

Unitization is applicable to multimodal cases in other ways. Just as there are misfires in unimodal unitization, there are misfires in multimodal unitization cases as well. In the unimodal case, you might see a face in a grilled cheese sandwich. Your perceptual system is unitizing something that is not in fact a face. Now consider the multimodal case of ventriloquism. Typically, when you see moving lips and hear a congruent sound, the sound comes from the lips. You have built up an association between moving lips and the sounds that come from them. In the ventriloquist effect, you see the dummy’s lips move, and you hear a congruent sound. Your perceptual system unitizes the dummy’s lips and the sound. Yet, this unitization is a misfire. The sound is not in fact coming from the dummy’s lips.

In many cases, unitization enables more efficient processing. Instead of having to see the jolt of the cymbal, hear the clang, and judge that they are both associated with the same object, the unitization process efficiently does this for you. It would take a longer time to have to see the jolt, hear the clang, and judge that they are part of the same object. Unitization is a way of embedding that task into our quick perceptual system. We get the same information—that the jolt and the clang are part of the same event—without having to make time-consuming inferences to get there. This frees up cognition to make other, more sophisticated, inferences. To draw an analogy, an elite tennis player might not have to think about her footwork because that task has been embedded into motor memory, freeing her mind to make more sophisticated judgments about what to do in the match. As in such cases of motor learning, unitization can free up cognition to do more sophisticated tasks.

The units involved in unitization may have complex internal structures. Think about the unitization of faces, for instance. The associations involved are not just between two or so elements, but can be quite complicated associations between various different features of a face. Multimodal associations might be complicated in a similar way. There may not just be simple associations between two elements, but rather complicated associations between various different multimodal features of a single object or event.

When Pourtois et al. (2000), Vatakis and Spence (2007), Kubovy and Schutz (2010), and Navarra et al. (2012) others assume that intermodal feature binding occurs, their background reasoning is perhaps something like the following. We know that intramodal binding occurs, that is, that features detected by a single sense modality get bound together, as when the shape and color of a cup get bound to it. We know that multimodal perception occurs. So we can take binding and extrapolate from the intramodal case to apply it to multimodal perception. The overall argument that I am making is structurally similar. We know that unitization occurs, and we know that multimodal perception occurs. So I am taking unitization and extrapolating from the unimodal case to apply it to multimodal perception. But how do we know which point is the right starting point? How do we know whether we should start with binding or start with unitization? I now want to turn to a few cases that I think are potentially difficult for the intermodal binding view to handle, but easy for the unitization view.

Start by considering a case of illusory lip-synching—a case where someone appears to be lip-synching, but is actually singing. Sometimes this might occur due to a mismatch in association between the audio and the visual. In 2009, for instance, a Scottish singer named Susan Boyle gained worldwide fame from her appearance on the TV show “Britain’s Got Talent.^6^” Her performance was captivating to many people because to them she did not look as if she could have such an impressive singing voice. They did not associate that sound with that look. And part of the good that came out of her case was that people broke their previous false association. Now imagine that you are in the audience as Susan Boyle steps on stage and sings. Plausibly, this would be a confusing experience. At first, you might not localize the sound at Susan Boyle’s moving lips. In your experience, it might be a case of illusory lip-synching. You might experience the sound as coming from elsewhere, even though it is actually coming from Susan Boyle.

Cases where vocal sounds are incongruous with the visual might be most vivid with pets, and amusing videos are often made documenting the results, showing animals that sound like human beings or like fire engine sirens. Consider one such example. Suppose you are listening to your radio with your dog nearby. A song comes on the radio that you haven’t heard before. You happen to glance over at your dog, who appears to be moving its mouth in synch with the vocals. Then you realize that what you thought were the vocals are actually coming from your dog^7^.

By appealing to learned associations, the singing dog case (and others like it) makes sense—the radio’s location and the dog’s sound get unitized. This happens because through experience, your perceptual system associates the sound that the dog makes with the radio. That sound is the kind of sound that would typically come from a radio. When the radio’s location and the dog’s sound get unitized, this is a misfire. The sound came from the dog and not from the radio. However, the misfire is understandable, given the fact that that type of sound typically comes from a radio and not a dog. We can apply the lesson of this case more generally. Past associations (between, say, types of sounds and types of things) determine the specific multimodal units that we experience.

It is unclear what psychologists who advocate intermodal feature binding would say about these sorts of cases. The dog’s mouth movement and the sound have happened at the exact same time, and from the same spatial location, but fail to be bound. But if binding were an automatic mechanism, wouldn’t intermodal binding just bind the dog’s voice to the dog’s mouth?

One option for the defender of binding is to hold that binding need not be automatic, but can be modulated by cognitive factors like whether or not the noise is the sound that a dog can make. For example, O’Callaghan (forthcoming, ms p. 15) quotes Vatakis and Spence (2007, p. 744), who claim that binding need not depend just on “low-level (i.e., stimulus-driven) factors, such as the spatial and temporal co-occurrence of the stimuli,” but can depend on “higher level (i.e., cognitive) factors, such as whether or not the participant assumes that the stimuli should ‘go together’.” If Vatakis and Spence (2007) and O’Callaghan (forthcoming) are right, then binding need not be automatic, since it can be modulated by cognitive factors.

If binding need not be automatic, but can be modulated by cognitive factors, then this presents a difficult challenge. My claim was that a view on which binding is automatic gets cases like the dog case wrong, since it would predict that the dog’s voice gets bound to the dog’s mouth, which is not what happens. Yet, if theorists defending an intermodal binding process can just weaken the automaticity requirement, then it seems that they can accommodate cases like the dog case into their model. One possible response is to appeal to parsimony. Given that it is difficult empirically to pull apart the associative account from the intermodal binding account, an appeal to the theoretical virtues of each view is warranted. If an associative view can handle all putative cases of intermodal binding, but an intermodal binding view cannot handle all cases without appealing to a learning mechanism (to deal with cases of involving the plausibility of combination), then it seems like parsimony supports the associative view. Of course, there may be other theoretical virtues to take into account when examining both views, as well as other empirical considerations, but it seems at the very least that parsimony tells in favor of an associative account.

## OBJECTIONS AND RESPONSES

My claim is that appealing to learned associations (such as the association between the dog’s sound and the radio’s location) makes sense of cases like the dog case. But one might object that there are other equally good or better ways of making sense of such cases. One alternative is that the singing dog case is just a straightforward crossmodal illusion, like the ventriloquist effect^8^. The idea is that just as in the ventriloquist effect, the auditory location of the sound gets bound to the moving lips, so too in the singing dog case, the sound gets bound to the location of the radio. In both cases, the experience is illusory. Just as the sound is not coming from the ventriloquist dummy in the ventriloquist case, so too is it not coming from the radio in the singing dog case.

My response is as follows. In the ventriloquist effect, both binding and association are viable explanations, at least on its face. For the associative explanation, it could be that we build an association between the sound of a voice and the movement of lips. On the other hand, an explanation just in terms of binding is equally plausible. It could be that we bind sounds with congruent movements together. In the singing dog case, however, only an explanation in terms of association will suffice. The associative explanation is that we build an association between voice sounds and radios, and so when the dog makes a voice sound, that sound gets unitized with the radio. An explanation just in terms of binding gives the wrong prediction for the singing dog case. If we bind sounds with congruent movements together, then the dog’s sound should be bound to the congruent movement of the dog’s mouth.

Consider a second objection that there is another equally good or better way of making sense of cases like the singing dog case. According to this objection, feature binding can be guided by categorical perception. The idea is that in the singing dog case, and cases like it, the categories that you have (of dog voices and radio sounds, for instance) influence what gets bound to what. So, there is a story to be told about the selection of features with regard to which features get bound together. And it is natural to suppose that categorical learning might have a role to play in which features get selected and thus bound together. Traditionally, the literature on binding has been very much concerned with sensory primitives like colors and shapes, and there’s a question about whether higher-level perceptual features get bound in that same way. According to this objection, we do not need to choose between feature binding and learned associations because they can play a role together^9^.

I find this objection to be plausible, yet currently unsubstantiated. To the best of my knowledge at least, there is no empirical evidence demonstrating the claim that categorical perception can guide feature binding. I take it to still be a plausible hypothesis, however, because there is some evidence that learning connections between sensory primitives can influence the binding process (Colzato et al., 2006). But as far as I know, this same influence has not been demonstrated for higher-level perceptual features. The objection is right in that it remains a live option that feature binding can be guided by categorical perception. Still, if the goal of the objection is to establish that there is another equally good or better way of making sense of cases like the singing dog case, in absence of empirical evidence to ground this alternative, the alternative is not a better explanation. There is empirical evidence, due to studies on unitization, to ground the explanation of the singing dog case in terms of learned association. So, barring empirical evidence to ground the explanation of that case in terms of categorical perception guiding feature binding, this explanation is not equal or better than the explanation in terms of learned association.

A third objection is to the idea that unitization can explain multimodal cases. According to this objection, unitization implies that there was something there before to unitize. But in certain cases of multimodal perception, this seems implausible. Take the case of flavor perception. Flavor is a combination of taste, touch, and retronasal (inward-directed) smell (see Smith, 2013). Yet, flavors are always just given to you as single unified perceptions. You are never given just the parts. You don’t start by having a retronasal smell experience, taste and touch, and then unitize those things^10^.

I think this objection points to an exception to the argument that I am making. Flavor perception is a special case of multimodal perception where a unitization account does not apply. This might seem *ad hoc*, but at the same time, it is well-recognized that flavor perception is a special case of multimodal perception in general. Flavor is special, because as O’Callaghan points out, it is a “type of feature whose instances are perceptible only multimodally” (O’Callaghan, 2014, ms p. 26). That is to say, where in the cymbal case, one can experience the jolt and the clang either together or separately (if one were to close one’s eyes or shut one’s hears, for instance), in the case of flavor properties, they are perceptible only through taste, touch, and retronasal smell. Given that, it should not be surprising that flavor has a special treatment.

A fourth objection continues on the third, but focuses on speech perception rather than flavor perception. According to this objection, there are documented cases of infant speech perception where an infant has a coupling without ever being exposed to either of the coupling’s components. For instance, before eleven months, Spanish infants can match /ba/ and /va/ sounds with corresponding images of someone unambiguously saying /ba/ and /va/ (Pons et al., 2009). Spanish itself does not make a distinction between /ba/ and /va/. Even if an infant is not surrounded by English speakers, for example, the infant before eleven months can still match audio and visual English phonemes. But how can this be through association when the infant herself was not surrounded by English speakers? Why are infants able to match the sounds with the images, and how can an associative account explain it?^11^.

This objection presents a difficult but not insurmountable challenge for the unitization view of multimodal perception. In the study in question (Pons et al., 2009), all infants initially underwent two 21 s trials in which they were presented with silent video clips of a bilingual speaker of Spanish and English, repeatedly producing a /ba/ syllable on one side of the screen and a /va/ syllable on the other side. So, while it is right to say that the Spanish infants had not been surrounded by English speakers, they had been exposed to English speakers. And it remains a possibility that this exposure was sufficient for matching audio and visual English phonemes through association. This possibility is more plausible if we allow that some pairs are more easily unitized than others, in this case /ba/ and /va/ sounds with corresponding images of someone unambiguously saying /ba/ and /va/.

## CONCLUSION

My account sides with O’Callaghan in one respect, and against the dominant view in psychology in another respect. With O’Callaghan, I accept that perceptual awareness is not just “minimally multimodal.” It is not just exhausted by perceptual awareness in each of the sense modalities happening at the same time. The cymbal case shows this. There is something that it is like to be aware that the jolt of the cymbal and the clang are part of the same event. And this is different from what it is like to just see the jolt and hear the clang. In holding this view, I depart from Spence and Bayne (2014), who find it debatable that it is part of one’s experience that the jolt and the clang are part of the same event, rather than part of post-perceptual processing or some kind of inference the subject makes.

According to the dominant view in psychology (including Pourtois et al., 2000; Vatakis and Spence, 2007; Kubovy and Schutz, 2010), multimodal experiences result from an intermodal feature binding process. Against this dominant view, however, I am a skeptic of intermodal feature binding. This is because I think that an associative process rather than a binding mechanism best explains multimodal perceptions. To show this, I outlined a specific associative process in the literature on perceptual learning that can explain multimodal perceptions: unitization. I argued, for instance, that unitization best explains what it is like to be aware that the jolt of the cymbal and the clang are part of the same event. The jolt and the clang are unitized in that event. So, I am skeptical of an explanation of this case, and cases like it, in terms of intermodal feature binding. Such multimodal perceptions are unitized, not bound^12^.

## Conflict of Interest Statement

The author declare that the research was conducted in the absence of any commercial or financial relationships that could be construed as a potential conflict of interest.

## Footnotes

1 O’Callaghan adds to this list: Bushara et al. (2003), Bertelson and de Gelder (2004), Spence and Driver (2004), Spence (2007), and Stein (2012).

2 Thanks to a reviewer for extensive suggestions about related literature, and the connections between that literature and the claims I make in the paper.

3 I thank Casey O’Callaghan and Diana Raffman for clarifying the relationship between O’Callaghan’s position and my own.

4 I will be drawing very closely from Goldstone (1998, pp. 602–604) and Goldstone and Byrge (2014) in explaining unitization.

5 Thanks to a reviewer for the symbolism.

6 For a video of her initial performance, which has been viewed over 150 million times, see: http://www.youtube.com/watch?v=RxPZh4AnWyk

7 As an example of this, see: https://www.youtube.com/watch?v=KECwXu6qz8I

8 Thanks to Casey O’Callaghan for raising this possibility.

9 Thanks to Tim Bayne for this objection.

10 I owe this objection to Barry C. Smith.

11 I owe this objection to Barry C. Smith and Janet Werker.

12 Thanks to Casey O’Callaghan, Tim Bayne, Barry C. Smith, Diana Raffman, Ophelia Deroy, Mohan Matthen, Peter Baumann, Janet Werker, Matthew Fulkerson, Adrienne Prettyman, Zoe Jenkin, Aaron Henry, an anonymous reviewer, and to the audience at the 2014 workshop on multisensory integration at the University of Toronto.
